# Gypenosides alleviates MCT-induced pulmonary arterial hypertension in rats by targeting oxidative stress, inflammation, and apoptosis

**DOI:** 10.22038/ijbms.2025.82437.17822

**Published:** 2025

**Authors:** Dan Du, Xiao-Wei Gong, Ya-Dong Yuan

**Affiliations:** 1 Department of Respiratory and Critical Care Medicine, The Second Hospital of Hebei Medical University, Shijiazhuang, 050000, China

**Keywords:** Apoptosis, Gypenosides, Inflammation, Monocrotaline, Oxidative stress, Pulmonary arterial - hypertension

## Abstract

**Objective(s):**

Pulmonary arterial hypertension (PAH) is a severe heart-lung condition characterized by complex changes in the pulmonary vasculature, known as pulmonary vascular remodeling (PVR). Gypenosides (Gyp) possesses a range of pharmacological properties, including anti-inflammatory and anti-oxidant effects. This study aims to explore the impact of Gyp on pulmonary vascular remodeling, particularly in relation to its potential to counteract inflammation, oxidative stress, and apoptosis.

**Materials and Methods:**

Twenty-four rats were randomly divided into four groups. MCT was administered via intraperitoneal injection at a 55 mg/kg dose to establish a PAH model. Gyp (150 mg/kg/day, Ig) was administered for 28 days, after which all lung tissues from the rats were isolated.

**Results:**

The findings indicated that Gyp had a substantial positive impact on the hemodynamics of rats with PAH induced by MCT, including reductions in mean pulmonary artery pressure (mPAP) and right ventricular systolic pressure (RVSP). Additionally, it exhibited inhibitory effects on right ventricular hypertrophy and pulmonary vascular remodeling in these PAH-afflicted rats. MCT elevated the concentration of MDA (*P*<0.01 in the lung) while reducing the levels of SOD and GSH-PX (*P*<0.0001). Furthermore, MCT enhanced the expression of IL-6, TNF-α, and IL-1β (*P*<0.0001), as well as the mRNA expression of NF-κB (*P*<0.001) and the Bcl2 level (*P*<0.0001), while it lowered the expression of Bax (*P*<0.0001). Conversely, Gyp treatment effectively mitigated all of these alterations.

**Conclusion:**

This study represents the initial investigation showing that Gyp treatment attenuates PVR by inhibiting oxidative stress, inflammation, and apoptosis, providing a foundation for further research.

## Introduction

Pulmonary arterial hypertension (PAH) is a progressive cardiopulmonary disease characterized by a mean pulmonary arterial pressure (mPAP) exceeding 20 mmHg and a pulmonary vascular resistance (PVR) of at least 2 Wood units (1, 2). It has been demonstrated in fundamental studies that the primary pathophysiological basis for the development of PAH is pulmonary vascular remodeling, and this process encompasses endothelial cell injury, over-proliferation of smooth muscle cells, resistance to apoptosis as well as infiltration by inflammatory cells (3, 4). The development of PAH is intricate; nonetheless, our comprehension of it is still insufficient. Given the seriousness of this condition and the absence of effective treatment alternatives, individuals suffering from PAH urgently need therapeutic approaches to alleviate and handle the disease.

Although the etiology of PAH is mostly unknown, there is growing recognition that both experimental and human PAH are involved in both initial and inflammatory processes. Inflammation is responsible for developing pulmonary artery smooth muscle (PASMC), which is critical for initiating and maintaining heart vessel reconstruction (5, 6). The levels of interleukin-1β (IL-1β), IL-6, tumor necrosis factor-α (TNF-α), and nuclear factor Kappa-B (NF-κB) have been altered in the lung of PAH, some of which may be related to the disease severity and prognosis (7). Among the proinflammatory signaling pathways, NF-κB plays an important role (8, 9). 

An earlier study demonstrated that oxidative stress (OS) plays a significant role in pulmonary arterial hypertension (PAH) pathophysiology. After the onset of PAH, there is an overproduction of reactive oxygen species (ROS) within the body, resulting in harm to a range of cellular macromolecules. This harm disrupts cellular signaling pathways and eventually leads to apoptosis of the cells. The inactivation and removal of ROS are facilitated by enzymes such as superoxide dismutase (SOD), glutathione peroxidase (GSH-PX), and catalase (CAT), which provide a degree of protection against free radical damage. Malondialdehyde (MDA), a final product of membrane lipid peroxidation, serves as a marker for the severity of oxidative stress and cellular damage. Consequently, the original characteristics and functions of proteins and nucleic acids are compromised, further exacerbating cellular injury (10).

Gypenosides (Gyp), derived from the traditional Chinese herb *Gynostemma pentaphyllum (Thunb.) Makino* exhibits various pharmacological effects. These include the regulation of lipid metabolism (11), prevention of atherosclerosis (12), anticancer (13), anti-inflammatory (14), antidiabetic (15), and anti-NAFLD properties (16), prevalent in numerous Asian nations as well as in the United States (17). By inhibiting NF-κB signaling pathways, Gyp may significantly obstruct the activation of internal inflammatory components, establishing it as a potential therapeutic choice for treating inflammatory disorders. For example, Gyp has been shown to safeguard cardiomyocytes from injury caused by ischemia/reperfusion (I/R), and this protective effect is mediated through the inhibition of NF-κB p65 activation via the MAPK signaling pathway (18). In addition, Gyp also provides a protective effect on retinal pigment epithelial (RPE) cells against oxidative damage (19). However, the function of Gyp in PAH remains ambiguous. Therefore, with the intricate relationship between these processes, Gyp shows promise for treating PAH in humans because of its anti-inflammatory and anti-oxidant properties, supported by biochemical, hemodynamic, and histopathological studies.

## Materials and Methods

### Materials

Urethane (20%) was purchased from Shanghai Chemical Reagent Co. (China). Gyp was obtained from Yuan Ye Biotechnology Co. (China). MCT and Sildenafil were obtained from MedChemExpress LLC(USA). The ELISA kit (TNF-α, IL-1β, and IL-6) was obtained from MultiSciences Co. (China). Moreover, the kit (RNA extraction and cDNA synthesis), the housekeeping and target primers, along with the SYBR qPCR Mix Kit, were acquired from Wuhan Servicebio Technology Co. (China).

### Animal materials

Male Sprague-Dawley rats, aged 7 weeks (weighing 200–250 g), were acquired from the animal facility at the second hospital of Hebei Medical University. The rats were kept in controlled conditions with a 12/12 hr light-dark cycle, suitable temperature and humidity, and allowed easy access to food and water. Ethical guidelines were adhered to throughout the experiments. The procedural details were endorsed by the ethical review committee at the Second Hospital of Hebei Medical University under the Animal Experiments Welfare and Ethics Resolution (No. 2023-AE078).

### Treatment of rats

A single subcutaneous injection of 55 mg/kg of body weight was administered for MCT treatment. All experimental groups were kept under comparable conditions for 28 days. The rats were randomly divided into four groups (n=6): 1) Control: received no intervention. 2) MCT: received MCT (55 mg/kg, first day, IP) .3) MCT+ Sildenafil: received MCT (55 mg/kg, first day, IP) + Sildenafil (20 mg/kg, from the second day, Ig) .4) MCT+ Gypenosides: received MCT (55 mg/kg, first day, IP) + Gypenosides (150 mg/kg, from the second day, Ig).

### Hemodynamic measurements

After 28 days, all rats’ body weight (BW) was measured, followed by urethane anesthesia at 1g/kg body weight via intraperitoneal injection. Subsequently, a PE catheter, preloaded with heparin, was introduced into the right ventricular systolic pressure (RVSP) measurement and extended to the pulmonary artery. The PowerLab system (AD Instruments) was utilized to document the mean pulmonary artery pressure (mPAP) and the right ventricular systolic pressure (RVSP) (20, 21). After hemodynamic measurements, lung tissue was removed for protein isolation and histological evaluation. The right ventricular hypertrophy index (RVHI) was expressed as the weight ratio of the right ventricle to the left ventricle and the interventricular septum (RV/LV+S).

### Protein analysis for ELISA

A sample of lung tissue was obtained, and the supernatant was removed. The concentrations of TNF-α, IL-1β, and IL-6 in rat lung tissues were then determined following the manufacturer’s instructions.

### Determination of oxidative stress

MDA, GSH-PX, and SOD levels in the lung were determined provided by the Nanjing Jiancheng Biological Engineering Institute.

### Hematoxylin-eosin (HE) staining

Saline was infused into the lung tissue via the pulmonary artery. For 24 hr, the left lung tissue was preserved in 4% paraformaldehyde, subsequently embedded in paraffin, and then sliced into sections. The morphological changes observed in the right ventricle and the pulmonary vessels were evaluated by applying HE staining. The vessel thickness calculation was performed as follows: the ratio of the inner diameter to the outer diameter of the pulmonary arterioles and the percentage of wall area (WA%) = [(total area - lumen area)/total area] ×100% was used to assess pulmonary vascular remodeling. The formula for determining the thickness of the pulmonary artery wall (WT) is WT (%) = (vessel wall thickness/external diameter) × 100% (22).

### Immunohistochemistry

Lung tissue sections embedded in paraffin (4 µm) underwent a de-waxing and hydration process, followed by washing with PBS (pH 7.2–7.4). After performing antigen retrieval at 100 °C and blocking with 5% BSA at room temperature for one hour, the sections were incubated overnight at 4 °C with a 1:500 dilution of α-SMA antibody (GB111364).

### Real-time quantitative PCR

As previously reviewed in detail **(**23**)**, total RNA was isolated from the lungs of rats using the TRIzol reagent. An RNA-to-cDNA first-strand reverse transcription kit was then employed to synthesize cDNA from the extracted RNA. Subsequently, real-time PCR was conducted, and the primer sequences for the genes are as follows:

β-Actin (forward: 5′-TGCTATGTTGCCCTAGACTTCG-3′and reverse: 5′-GTTGGCATAGAGGTCTTTACGG- 3′, product size, 240 bp, NM_031144); NF-κB p65 (forward: 5′- CAGATACCACTAAGACGCACCC-3′ and reverse: 5′- CTCCAGGTCTCGCTTCTTCACA -3′, product size, 227 bp, NM_199267.2.).

### Western blotting

The proteins were extracted and quantified using a BCA detection kit. Following separation on 10% SDS-polyacrylamide gels, the proteins were electrophoretically transferred onto PVDF membranes**(**24**)**. The overnight exposure of membranes, which were impeded by 5% Bovine Serum Albumin to anti-Bcl-2 (GB154830, 1:1000; Servicebio), anti-Bax (GB15690, 1:1000; Servicebio), or β-Actin antibody (GB15003, 1:5000; Servicebio), was implemented at 4 °C after PVDF membranes were incubated with anti-rabbit IgG-HRP(SA00001, 1:10000; Proteintech), the analysis was conducted with the application of Fiji ImageJ-win64 software.

### Statistical analysis

The results were analyzed using GraphPad Prism 8, and the data are shown as the mean ± standard deviation (SD). One-way analysis of variance (ANOVA) was employed for the statistical evaluation, considering a *P*-value<0.05 as significant. Additionally, Tukey’s test was applied for pairwise comparisons.

## Results

### mPAP analysis

**Figure 1:**
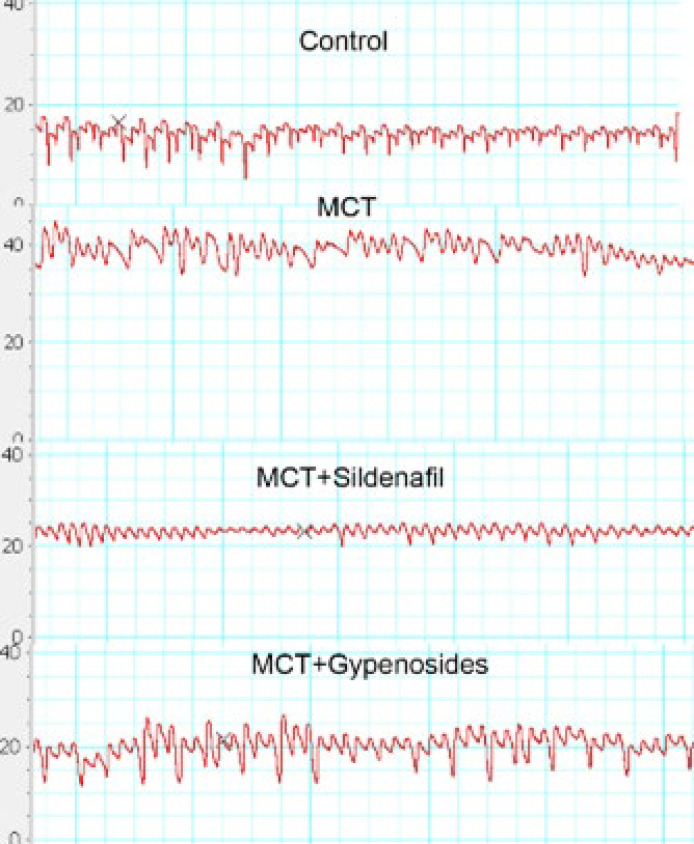
Effects of gypenosides treatment on PAH in male SD rats (n = 6)

PAH: Pulmonary arterial hypertension; MCT: Monocrotaline; Gyp: Gypenosides

Figure 1 shows the pressure curves of the rat pulmonary artery recorded by a PowerLab physiological recorder; Gyp positively influenced the aforementioned factors related to complications arising from the PAH model.

### Evaluation of hemodynamic functions and RVHI measurement

**Figure 2:**
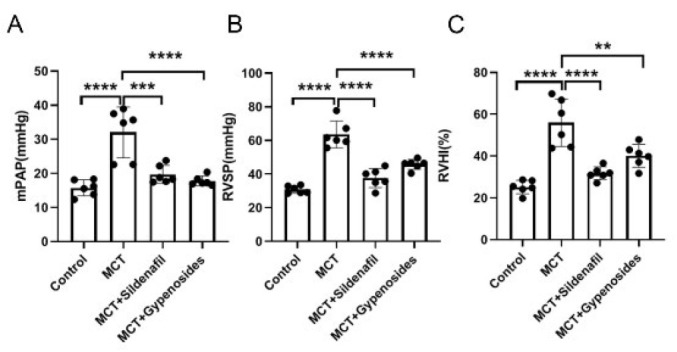
Effect of Gyp on hemodynamics in male SD rats

As demonstrated in Figure 2 and Table 1, the mPAP and RVSP observed in the MCT group were markedly elevated compared to those in the control group (*P*<0.0001). Additionally, the subjects in the positive group and those receiving MCT combined with Gyp declared significantly lower mPAP and RVSP levels when contrasted with the PAH group(*P*<0.001). A notable rise in the Fulton index, which serves as a measure of right ventricular hypertrophy, was noted in the PAH group (*P*<0.001). In contrast to the model group, Gyp decreased the cardiac hypertrophy index considerably.

### Gyp inhibits lung tissue inflammation cytokines

**Figure 3:**
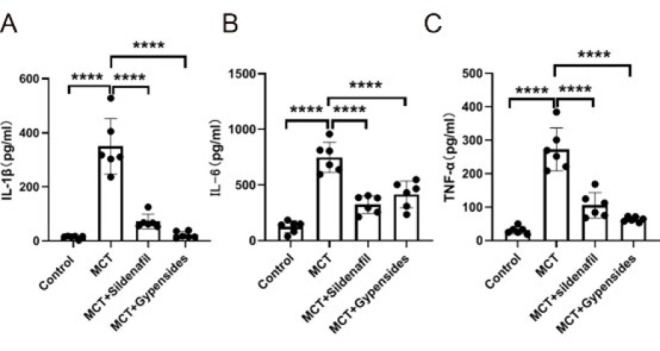
Gyp inhibits lung tissue inflammation cytokines in male SD rats

The expression of inflammatory cytokines was investigated in the lungs of model rats through ELISA. This analysis revealed a notable elevation in the protein levels of key inflammatory markers, specifically IL-6, IL-1β, and TNF-α, in the MCT group compared to the control group, with a statistical significance determined by *P*<0.01. Furthermore, treatment with Gyp resulted in a substantial reduction in the protein levels of these three inflammatory cytokines that were elevated due to MCT induction, with the results again reaching a level of statistical significance (*P*<0.01) (Figure 3).

### Effect of co-administration of Gyp and MCT on NF-κB p65 in lung tissue

**Figure 4:**
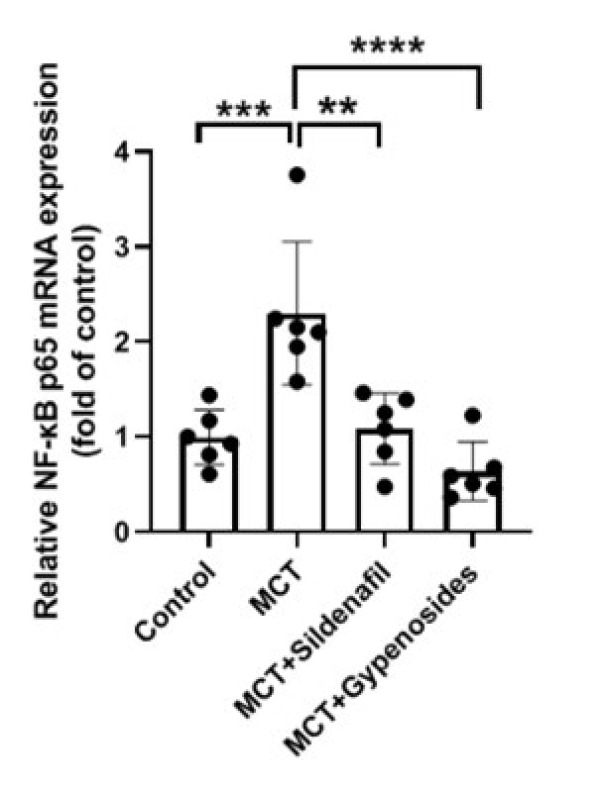
RT-qPCR was utilized for assessing NF-κB p65 mRNA expression in male SD rats

Administration of MCT resulted in a notable increase in NF-κB p65 levels, reaching a concentration of 2.30±0.75 ng/ml. This measurement was remarkably higher when compared to the control group, which exhibited NF-κB p65 levels of 0.99±0.29 ng/ml. These two groups’ differences were statistically significant, with a *P*-value less than 0.001. In contrast, the Gyp treatment substantially reduced NF-κB p65 levels within the MCT+Gypenosides group, where the levels decreased to 0.64±0.31 ng/ml. This data analysis reveals that Gyp may serve a purpose in mitigating the elevated NF-κB p65 levels induced by MCT administration in lung tissue (Figure 4).

### Effect of co-administration of Gyp and MCT on MDA, GSH-PX, and SOD in lung tissue

**Figure 5:**
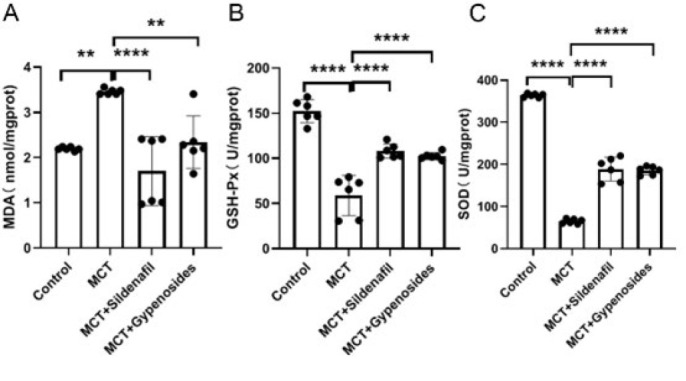
Oxidative stress markers in male SD rats with Monocrotaline (MCT)-induced pulmonary hypertension

The administration of MCT notably elevated the levels of MDA in lung tissue, reaching 3.46±0.06 nmol/mg protein. This result was remarkably higher than that observed in the control group, which recorded MDA levels of 2.19±0.04 nmol/mg protein, with a statistically significant difference indicated by* P*<0.01. Conversely, when Gyp was co-administered, it reduced MDA levels, bringing them down to 2.34±0.58 nmol/mg protein, further supporting the beneficial impact of Gyp in mitigating MCT-induced oxidative stress. Additionally, the levels of GSH-PX in lung tissue were significantly decreased following MCT administration, measuring at 58.85±22.07 U/mg protein. This starkly contrasted with the control group, which exhibited a much higher activity level of 152.33±12.71 U/mg protein, with a highly significant difference (*P*<0.0001). Notably, the concurrent treatment with Gyp was effective in partially reversing the reduction in GSH-PX levels, resulting in an increase to 102.48±4.01 U/mg protein, reinforcing the protective role of Gyp in maintaining anti-oxidant enzyme activity. Furthermore, the levels of SOD in lung tissue were also significantly diminished after MCT administration, with recorded values of 65.08±5.63 U/mg protein. In contrast, the control group exhibited a normal range of SOD levels at 363.85±4.37 U/mg protein, demonstrating a significant reduction with *P*<0.0001. However, co-treatment with Gyp led to a considerable restoration of SOD levels, which were measured at 185.66±9.92 U/mg protein, thus highlighting Gyp’s potential to enhance the anti-oxidant defenses in lung tissue in the context of MCT exposure (Figure 5).

### Histology of lung tissue

**Figure 6:**
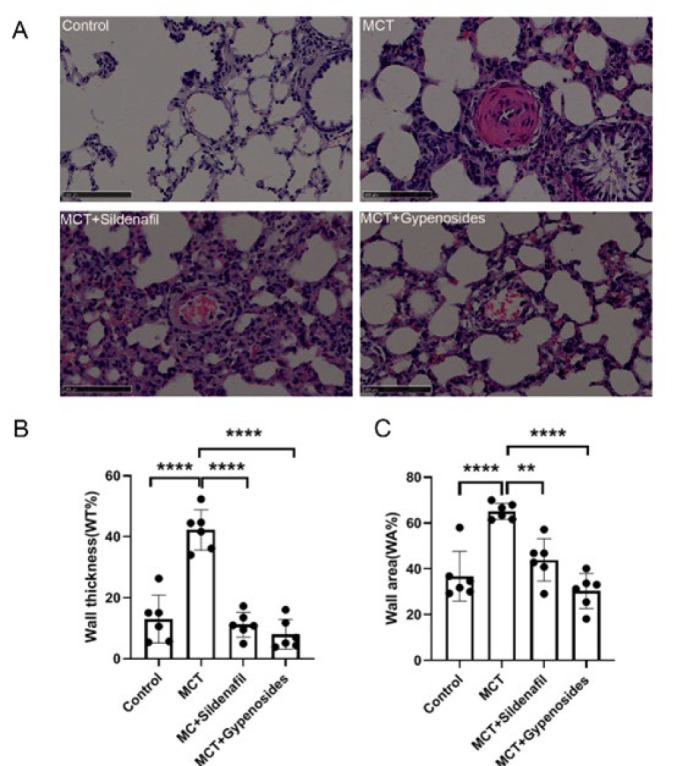
Gyp improves pathological changes of the pulmonary vasculature in male SD rats

After MCT treatment for 28 days, we performed H&E staining to determine pulmonary vascular remodeling. H&E staining was used to assess pulmonary vascular remodeling. The thickness of the pulmonary artery tube wall in the PAH group was notably more significant than that observed in control, Sildenafil, and Gyp groups, which were associated with a reduction in these histopathological alterations compareed to the PAH group(all *P*<0.05) (Figure 6).

### Histology of heart tissue

**Figure 7:**
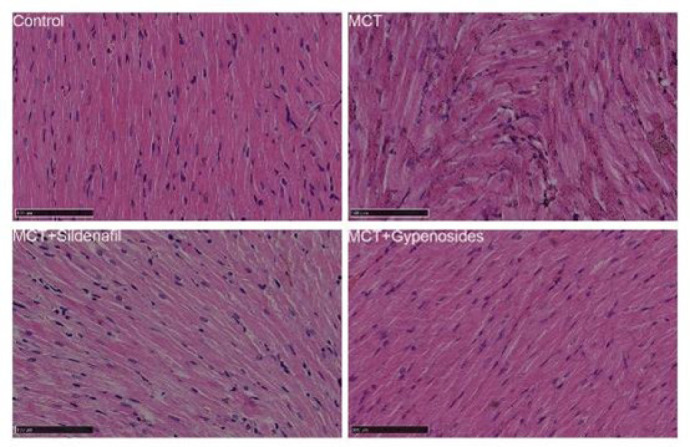
Gyp improves pathological changes in the heart in male SD rats

H&E staining of RV tissue showed that the cardiomyocytes in the control group had good continuity, regular arrangement, and small gaps. At the same time, compared with the control group, the cardiomyocytes of rats injected with MCT showed obvious proliferation and hypertrophy, disordered arrangement, cytoplasmic swelling, and increased cardiomyocyte gaps. Compared with MCT rats, Gyp-treated rats showed significant improvements in all of the above aspects (Figure 7).

### Effects of Gyp on α-SMA expression

**Figure 8:**
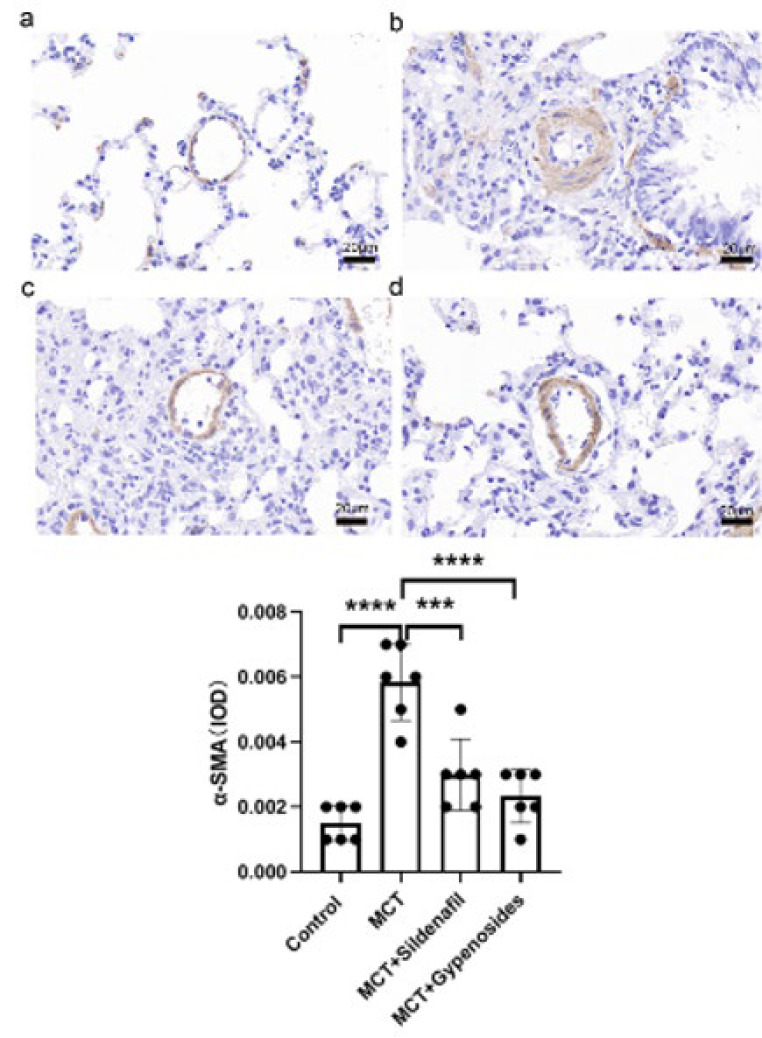
Effects of Gyp on α-SMA expression in male SD rats

Gyp has been explained to reduce the over-proliferation of PASMCs induced by MCT effectively. To investigate this phenomenon, immunohistochemistry staining was employed to analyze the expression levels of α-SMA within lung tissues obtained from rats. The findings indicated a marked increase in α-SMA expression in the MCT-treated group compared to those in the control group, underscoring the impact of MCT on smooth muscle cell proliferation. (*P*<0.01; Figure 8).

### Effect of Gyp on the expression of Bax and Bcl-2 in the lung of rats by MCT administration

**Figure 9:**
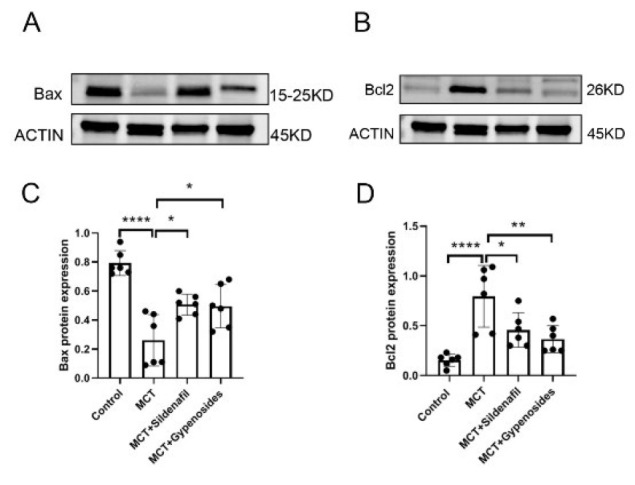
Effect of Gyp on the Western blot in the lung of male SD rats

In rats given MCT, the level of Bax expression was significantly elevated in the lungs compared to the control group (*P*<0.0001). Nevertheless, for the positive and Gyp group in the follow-up, the Bax expression in the MCT rats showed a decrease relative to the PAH group (*P*=0.0168 vs *P*=0.0226) (Figure 9). Moreover, the injection of MCT led to a notable increase in Bcl-2 expression in the lungs of the rats (*P*<0.0001) when juxtaposed with the control group. Treatment with Gyp inhibited the decline of Bcl-2 expression in the lungs of rats that had received MCT injections (*P*=0.0049) (Figure 9). It is essential to highlight that the levels of both Bax and Bcl-2 in the rats’ lungs exhibited significant variations compared to those in the control group (Figure 9).

### Mortality rate

In both the control group and those receiving monocrotaline injections, all rats lived throughout the duration of the study. The positive group also experienced the loss of two animals during the three-week period. Conversely, Gyp treatment fully averted any fatalities. To maintain a total of six animals in each group by the study’s conclusion (sampling time), deceased animals were substituted with new ones.

## Discussion

Pulmonary hypertension is characterized by hemodynamic changes in the pulmonary arteries. These changes lead to a significant increase in RVSP and mPAP in PAH. Our study showed that treatment with Gyp for 28 days improved mPAP, RVSP, and RV/(LV+S) weight ratio. We also demonstrated that these improvements were associated with oxidative stress, inflammation, and apoptosis. 

The MCT-induced pulmonary hypertension (PH) model has been extensively utilized for almost six decades due to its ease of implementation, consistent reproducibility, and affordability. MCT must be converted into its toxic metabolite, MCT pyrrole (MCTP), by the liver enzyme cytochrome P450 3A4 (CYP3A4), which subsequently causes vascular endothelial cell (EC) damage and inflammatory responses(25). Typically, MCT-induced PH is demonstrated in rats following a single subcutaneous injection of MCT at a dosage of 50–80 mg/kg(26). Consequently, in line with this methodology, our study utilized an MCT-induced group to explore the therapeutic effects of Gyp.

### Gyp effects on hemodynamic factors

Previous research has indicated that hemodynamic indicators such as mPAP, RVSP, and RVHI are significantly elevated in animal models of pulmonary hypertension. Consequently, the present study examined the impact of Gyp on the hemodynamic parameters in rats with MCT-induced PAH. The findings revealed that Gyp can mitigate the abnormal rise in mPAP and RVSP levels in PAH-affected rats, implying that Gyp may help lower pulmonary arterial pressure. Furthermore, PVR is a crucial pathophysiological characteristic of PAH, encompassing thickening of the pulmonary artery walls, muscular alterations, adventitial fibrosis, and eventually leading to right heart failure (27). Prior research frequently assessed pulmonary vascular remodeling and right ventricular hypertrophy through the examination of indicators such as wall thickness percentage (WT%), wall area percentage (WA%), and right ventricular hypertrophy index (RVHI). The findings from this investigation revealed that Gyp significantly decreased WT%, WA%, and RVHI in rats with PAH, indicating that Gyp has notable effects on both PVR and right ventricular hypertrophy in this model.

### Immunohistochemical staining was used to determine α-SMA in rat lung

Gyp has been observed to have protective effects against PAH by inhibiting cellular proliferation. This suggests that Gyp may play a crucial role in modulating the growth of PASMCs, thereby offering a potential therapeutic benefit. One of the main pathological changes responsible for the increase in pulmonary vascular resistance is the remodeling of pulmonary arterioles, primarily resulting from the excessive proliferation of PASMCs (28). Our current study evaluated the expression of α-SMA, a well-known marker for PASMCs, within rat lung tissues. Our results showed that MCT-induced expression of α-SMA was notably diminished when Gyp was present.

### Gyp effects on the levels of the inflammatory index in lung tissue

Inflammation plays a crucial role in vascular remodeling in both animal models of PAH and humans (29). Therefore, the reduction of inflammatory response may alleviate the occurrence of PAH (30). Moreover, Hong J conducted single-cell RNA sequencing on PAH model rats, revealing significant activation of the NF-κB pathway across various cell types (31). The role of NF-κB in the pathogenesis of PAH is well-documented and underscores its critical position in the disease’s development. Acting as a nuclear transcription factor, NF-κB modulates numerous proinflammatory genes, including TNF-α, IL-1β, and IL-6, which are instrumental in driving perivascular inflammation, the proliferation of PASMC, and ultimately the onset of PAH (32, 33). Prior research has indicated that IMD-0354 alleviates PAH through NF-κB inhibition (34). Supporting these results, our current study also demonstrated that Gyp suppressed NF-κB activation, downregulated the expression of IL-1β and IL-6, and ultimately attenuated PASMC proliferation. Consequently, we propose that Gyp alleviates PAH by blocking the NF-κB signaling pathway, which in turn diminishes vascular remodeling.

### Gyp effects on the oxidative stress of rat lung tissue

SOD is a vital anti-oxidant enzyme that plays a crucial role in neutralizing superoxide anions, thereby providing essential protection against oxidative damage and inflammatory responses. The function of SOD is particularly important within biological systems, as it contributes significantly to maintaining cellular integrity by mitigating the detrimental effects of oxidative stress(35). Additionally, GSH-PX and SOD together are instrumental in modulating reactive oxygen species (ROS) levels within the body(36). Conversely, MDA serves as a byproduct of lipid peroxidation occurring within cells, making its concentration an essential biomarker for assessing the extent of lipid peroxidation and overall oxidative stress experienced by the tissue. Elevated levels of MDA indicate a higher degree of oxidative damage, which is often implicated in various pathological conditions (37). In this context, Gyp has been shown to possess the capacity to inhibit oxidative stress across various diseases, corroborating our findings. Thus, the anti-oxidant properties of Gyp may also play a significant role in preventing pulmonary arterial remodeling, thereby contributing positively to vascular health.

### Gyp effects on apoptosis in rat lung tissue

It has been extensively documented that PAH is characterized by a notable resistance to programmed cell death, commonly called apoptosis (38). One significant contributor to this resistance appears to be the Warburg effect and the balance of mitochondrial fusion and fission (39). Apoptosis resistance is closely linked to tissue proliferation, particularly within vascular tissues, potentially playing a role in developing arterial hypertrophy in the lungs of patients with PAH. A shift in the balance between the pro-apoptotic protein Bax and the anti-apoptotic protein Bcl-2 often results in diminished apoptosis in pulmonary artery smooth muscle cells (PASMCs), worsening the condition (40). Our recent study unveiled a significant decrease in apoptosis within lung tissues, coupled with an increase in medial artery hypertrophy in the lungs after three weeks of treatment with MCT injection. This aligns with earlier observations in the field. Notably, our findings highlighted that treatment with Gyp, a specific agent, induced a pro-apoptotic effect, counteracting the elevated levels of apoptosis typically seen in MCT-induced PAH models. While the exact molecular mechanisms through which Gyp operates remain to be fully elucidated, these results are promising and suggest potential avenues for therapeutic intervention in PAH.

Results showed the mPAP, RVSP, and RVHI of the rats in the control, Gyp, and positive groups of rats. Results are expressed as mean ± SD (n=6). Significance levels were denoted as ***P<0.001, ****P<0.0001. mPAP: mean pulmonary arterial pressure; RVSP: Right ventricular systolic pressure; MCT: Monocrotaline; PAH: Pulmonary arterial hypertension

(A) Levels of IL-1β, (B) IL-6 levels in lung tissue, and (C) Levels of TNF-α.

mean±SD is shown in the results (n=6). Significance levels were denoted as ****P<0.0001.

MCT: Monocrotaline; Gyp: Gypenosides; IL-1β: Interleukin-1 beta; IL-6: Interleukin-6; TNF-α:Tumour necrosis factor-alpha

**Table 1:** Effects of Gyp on mPAP, RVSP, and RVHI measurement in male SD rats

Group	mPAP (mmHg)	RVSP (mmHg)	RVHI (%)
Control	15.77+2.35	30.82+2.21	25.24+3.21
MCT	32.05+7.44	63.65+7.90	57.09+12.35
MCT+Sildenafil	19.72+2.63	37.68+5.72	31.78+3.09
MCT+Gypenosides	17.80+1.41	45.79+3.00	40.18+5.45

mPAP: mean pulmonary arterial pressure; RVSP: Right ventricular systolic pressure; RVHI: Right ventricular hypertrophy index; MCT: Monocrotaline; Gyp: Gypenosides

Values are represented as means±standard deviation, n=6. A one-way ANOVA test was used, **P<0.01 and ****P<0.0001.

MCT: Monocrotaline; NF-κB:Nuclear factor kappa B p65

(A) MDA content. (B) GSH-PX activity. (C) SOD expression. Results are expressed as mean ±SD (n = 6). Significance levels were denoted as **P<0.01, ****P<0.0001.

MDA: Malondialdehyde; GSH-PX: Glutathione peroxidase; SOD: Superoxide dismutase

(A) Representative images of HE staining of each group (scale bar = 100 μm) were displayed; Magnification, ×400. (B and C) WT% and WA% index respectively (n=6). **P<0.01, ****P<0.0001. MCT: Monocrotaline; Gyp: Gypenosides; WT%, the ratio of the vascular walls thickness; WA%, the ratio of the vascular wall area

In the MCT group, there was a notable expansion of the intercellular substance. Furthermore, the myofibrils exhibited a disrupted and disordered arrangement, which highlights the extent of cellular disorganization. This disorganization is clearly illustrated in the provided figure. The magnification of the image is 400×. Gyp: Gypenosides; MCT: Monocrotaline

α-SMA analysis of different groups. Magnification, ×400, scale bar, 20µm. Optical density of α-SMA. ***P<0.001. ****P<0.0001. n=6. α-SMA: alpha-smooth muscle actin; Gyp: 150 mg/kg Gypenosides; MCT: Monocrotaline.

(A) Bax and (B) Bcl2.

Data are presented as mean± SD; n=6. *P<0.05, **P<0.01, and ****P<0.0001. Gyp: 150 mg/kg Gypenosides; MCT: Monocrotaline.

## Conclusion

The study generally discovered that MCT-induced PAH led to inflammation, oxidative stress, and anti-apoptosis in laboratory animals. Furthermore, the study showed that cardiovascular issues induced by PAH were prevented, and cardiac structural changes were protected by co-administration of Gyp, a natural anti-oxidant. Based on these results, we can infer that co-treatment with Gyp helps avert cardiac dysfunction linked to PAH by boosting factors that reduce inflammation, combat oxidative stress, and mitigate apoptosis.

## Data Availability

Data associated with the study have not been deposited into a publicly available repository but will be made available upon request.

## Declaration

We have not used any AI tools or technologies to prepare this manuscript.

## References

[1] Humbert M, Kovacs G, Hoeper MM, Badagliacca R, Berger RMF, Brida M (2023). 2022 ESC/ERS guidelines for the diagnosis and treatment of pulmonary hypertension. Eur Respir J.

[2] Kularatne M, Gerges C, Jevnikar M, Humbert M, Montani D (2024). Updated clinical classification and hemodynamic definitions of pulmonary hypertension and its clinical implications. J Cardiovasc Dev Dis.

[3] Humbert M, Guignabert C, Bonnet S, Dorfmüller P, Klinger JR, Nicolls MR (2019). Pathology and pathobiology of pulmonary hypertension: state of the art and research perspectives. Eur Respir J.

[4] Liu Y, Tang BL, Lu ML, Wang HX (2023). Astragaloside IV improves pulmonary arterial hypertension by increasing the expression of CCN1 and activating the ERK1/2 pathway. J Cell Mol Med.

[5] Wang RR, Yuan TY, Wang JM, Chen YC, Zhao JL, Li MT (2022). Immunity and inflammation in pulmonary arterial hypertension: From pathophysiology mechanisms to treatment perspective. Pharmacol Res.

[6] Stenmark KR, Frid MG, Graham BB, Tuder RM (2018). Dynamic and diverse changes in the functional properties of vascular smooth muscle cells in pulmonary hypertension. Cardiovasc Res.

[7] Zhao H, Song J, Li X, Xia Z, Wang Q, Fu J (2024). The role of immune cells and inflammation in pulmonary hypertension: Mechanisms and implications. Front Immunol.

[8] Huertas A, Tu L, Humbert M, Guignabert C (2020). Chronic inflammation within the vascular wall in pulmonary arterial hypertension: more than a spectator. Cardiovasc Res.

[9] Farkas D, Alhussaini AA, Kraskauskas D, Kraskauskiene V, Cool CD, Nicolls MR (2014). Nuclear factor κB inhibition reduces lung vascular lumen obliteration in severe pulmonary hypertension in rats. Am J Respir Cell Mol Biol.

[10] Chai Y, Gu X, Zhang H, Xu X, Chen L (2024). Phoenixin 20 ameliorates pulmonary arterial hypertension via inhibiting inflammation and oxidative stress. Aging (Albany NY).

[11] Xie P, Luo HT, Pei WJ, Xiao MY, Li FF, Gu YL (2024). Saponins derived from gynostemma pentaphyllum regulate triglyceride and cholesterol metabolism and the mechanisms: A review. J Ethnopharmacol.

[12] Huang YP, Wang YS, Liu YY, Jiang CH, Wang J, Jiang XY (2022). Chemical characterization and atherosclerosis alleviation effects of gypenosides from gynostemma pentaphyllum through ameliorating endothelial dysfunction via the PCSK9/LOX-1 pathway. J Agric Food Chem.

[13] Qi YS, Xie JB, Xie P, Duan Y, Ling YQ, Gu YL (2021). Uncovering the anti-NSCLC effects and mechanisms of gypenosides by metabolomics and network pharmacology analysis. J Ethnopharmacol.

[14] Tu Q, Zhu Y, Yuan Y, Guo L, Liu L, Yao L (2021). Gypenosides inhibit inflammatory response and apoptosis of endothelial and epithelial cells in LPS-induced ALI: A study based on bioinformatic analysis and in vivo/vitro experiments. Drug Des Devel Ther.

[15] Zhu KN, Tian SS, Wang H, Tian YS, Gu GZ, Qiu YY (2021). [Study on effect of gypenosides on insulin sensitivity of rats with diabetes mellitus via regulating NF-κB signaling pathway]. Zhongguo Zhong Yao Za Zhi.

[16] Zhou T, Cao L, Du Y, Qin L, Lu Y, Zhang Q (2023). Gypenosides ameliorate high-fat diet-induced nonalcoholic fatty liver disease in mice by regulating lipid metabolism. PeerJ.

[17] Su C, Li N, Ren R, Wang Y, Su X, Lu F (2021). Progress in the medicinal value, bioactive compounds, and pharmacological activities of Gynostemma pentaphyllum. Molecules.

[18] Yu H, Shi L, Qi G, Zhao S, Gao Y, Li Y (2016). Gypenoside protects cardiomyocytes against ischemia-reperfusion injury via the inhibition of mitogen-activated protein kinase mediated nuclear factor kappa B pathway in vitro and in vivo. Front Pharmacol.

[19] Alhasani RH, Biswas L, Tohari AM, Zhou X, Reilly J, He JF (2018). Gypenosides protect retinal pigment epithelium cells from oxidative stress. Food Chem Toxicol.

[20] Liu J, Fang G, Lan C, Qiu C, Yao L, Zhang Q (2024). Forsythoside B mitigates monocrotaline-induced pulmonary arterial hypertension via blocking the NF-κB signaling pathway to attenuate vascular remodeling. Drug Des Devel Ther.

[21] Sheng Y, Gong X, Zhao J, Liu Y, Yuan Y (2022). Effects of crocin on CCL2/CCR2 inflammatory pathway in monocrotaline-induced pulmonary arterial hypertension rats. Am J Chin Med.

[22] Gao AR, Li S, Tan XC, Huang T, Dong HJ, Xue R (2022). Xinyang tablet attenuates chronic hypoxia-induced right ventricular remodeling via inhibiting cardiomyocytes apoptosis. Chin Med.

[23] Yang M, Zhang T (2024). Genistein inhibits the release of proinflammatory substances from macrophages by suppressing potassium loss- and ROS-mediated caspase-1/gasdermin D pathway activation and pyroptotic cell lysis. Iran J Basic Med Sci.

[24] Aimaier S, Tao Y, Lei F, Yupeng Z, Wenhui S, Aikemu A (2023). Protective effects of the Terminalia bellirica tannin-induced Nrf2/HO-1 signaling pathway in rats with high-altitude pulmonary hypertension. BMC Complement Med Ther.

[25] Gomez-Arroyo JG, Farkas L, Alhussaini AA, Farkas D, Kraskauskas D, Voelkel NF (2012). The monocrotaline model of pulmonary hypertension in perspective. Am J Physiol Lung Cell Mol Physiol.

[26] Wu XH, Ma JL, Ding D, Ma YJ, Wei YP, Jing ZC (2022). Experimental animal models of pulmonary hypertension: Development and challenges. Animal Model Exp Med.

[27] Chen Y, Ma P, Bo L, Lv Y, Zhou W, Zhou R (2024). Isorhamnetin alleviates symptoms and inhibits oxidative stress levels in rats with pulmonary arterial hypertension. Iran J Basic Med Sci.

[28] Wang J, Wang L, Chen X, Liang ML, Wei DH, Cao W (2022). Cigarette smoke extract stimulates human pulmonary artery smooth muscle cell proliferation: Role of inflammation and oxidative stress. Iran J Basic Med Sci.

[29] Hu Y, Chi L, Kuebler WM, Goldenberg NM (2020). Perivascular inflammation in pulmonary arterial hypertension. Cells.

[30] Yoo HHB, Marin FL (2022). Treating inflammation associated with pulmonary hypertension: An overview of the literature. Int J Gen Med.

[31] Hong J, Arneson D, Umar S, Ruffenach G, Cunningham CM, Ahn IS (2021). Single-cell study of two rat models of pulmonary arterial hypertension reveals connections to human pathobiology and drug repositioning. Am J Respir Crit Care Med.

[32] Soon E, Holmes AM, Treacy CM, Doughty NJ, Southgate L, Machado RD (2010). Elevated levels of inflammatory cytokines predict survival in idiopathic and familial pulmonary arterial hypertension. Circulation.

[33] Savai R, Pullamsetti SS, Kolbe J, Bieniek E, Voswinckel R, Fink L (2012). Immune and inflammatory cell involvement in the pathology of idiopathic pulmonary arterial hypertension. Am J Respir Crit Care Med.

[34] Hosokawa S, Haraguchi G, Sasaki A, Arai H, Muto S, Itai A (2013). Pathophysiological roles of nuclear factor kappaB (NF-kB) in pulmonary arterial hypertension: Effects of synthetic selective NF-kB inhibitor IMD-0354. Cardiovasc Res.

[35] Yao W, Fan M, Qian H, Li Y, Wang L (2024). Quinoa polyphenol extract alleviates non-alcoholic fatty liver disease via inhibiting lipid accumulation, inflammation and oxidative stress. Nutrients.

[36] Kim SJ, Jo YJ, Jeong SH, Kim YH, Hee Han J (2024). An investigation of antioxidative and anti-inflammatory effects of Taraxacum coreanum (white dandelion) in lactating Holstein dairy cows. J Adv Vet Anim Res.

[37] Mokhtari T, Lu M, El-Kenawy AE (2023). Potential anxiolytic and antidepressant-like effects of luteolin in a chronic constriction injury rat model of neuropathic pain: Role of oxidative stress, neurotrophins, and inflammatory factors. Int Immunopharmacol.

[38] Jin H, Jiao Y, Guo L, Ma Y, Zhao R, Li X (2021). Astragaloside IV blocks monocrotalineinduced pulmonary arterial hypertension by improving inflammation and pulmonary artery remodeling. Int J Mol Med.

[39] Li X, Tan J, Wan J, Cheng B, Wang YH, Dai A (2024). Cell death in pulmonary arterial hypertension. Int J Med Sci.

[40] Lin J, Chen R, Liao H, Zhang Y, Zheng Z, Hong C (2024). Mechanisms of cordycepin in the treatment of pulmonary arterial hypertension in rats based on metabonomics and transcriptomics. Sci Rep.

